# Conversations upon the Physical and Mental Hygiene of Girlhood

**Published:** 1881-04-20

**Authors:** T. S. Powell

**Affiliations:** Atlanta; Professor of Obstetrics and Diseases of Women, and Lecturer on Medical Ethics in the Southern Medical College


### Southern Medical Record.

EDITORS:

T. S. POWELL, M.D. W. T. GOLDSMITH, M.D. R. C. WORD, M.D.

*R. C. WORD, M.D., Managing Editor.*

☞All Communications and Letters on Business connected with the RECORD must be addressed to the Managing Editor.

VOL. XI. ATLANTA, GA., APRIL 20, 1881. NO. 4.

### ORIGINAL AND SELECTED ARTICLES.

### CONVERSATIONS UPON THE PHYSICAL AND MENTAL HYGIENE OF GIRLHOOD.

BY T. S. POWELL, M. D.,

Professor of Obstetrics and Diseases of Women, and Lecturer on Medical Ethics in the Southern Medical College.

### CONVERSATION IV.

Mother—"Good morning, Doctor. I am glad you have called. I have just received a note from Mary, at her uncle's, and she is delighted with country life; she writes me that she is already feeling much better and stronger than when she left home, ten days ago."

Doctor—"Well, madam, I am glad to hear it, but am not surprised. If she follows directions she will continue to improve, and no doubt will return next fall in much better health."

Mother—"I really hope so. I will do all I can to bring it about, and as I will go out to see Mary two or three times during the summer, I will take any new directions from you, Doctor, and keep you informed of her condition."

Doctor—"Very well, madam, I will be glad to have you do so, and if any unfavorable change should occur in your daughter's case, I will myself go out to see her."

Mother—"I thank you, Doctor; you are so kind. I hope, however, it will not be necessary to give you that trouble. You are not in a great hurry this morning, are you? Say 'no,' Doctor, and let us begin our conversation just where we left off at your last visit."

Doctor—"Well, madam, I can give you one hour this morning. But I don't believe you remember where we ended our last conversation. If you will count them, you will see that it has been precisely eight days since then."

Mother—"Yes, Doctor, I know it has been just one week, and your visit did not supprise me this morning. I was thinking of you when the bell rang, and felt certain it was you, because I did not forget your last words, as they made an impression on my mind, which I have attempted to use for the benefit of my own family, and by which I think we have all profited greatly. I am trying to teach my children the importance of being punctual to engagements of every character, and that to do this is also a mark of good breeding. You remember there was a European monarch I believe, who said 'punctuality is the politeness of kings,' and I am sure it ought then to be of lesser mortals like ourselves."

Doctor—"I am sorry to know that I am not noted for punctuality at all times, but I can say that when I have thus failed to meet my engagements, I would not give a false excuse by saying I was pressed with other business, or something to that effect, but promptly told the truth, and said I forgot the engagement. A false excuse or apology for failure in punctuality often makes matters worse, as some remedies do diseases."

Mother—"Yes, I have frequently heard apologies given that were worse than the offence itself."

Doctor—"When I was about twenty-one years of age, I was much impressed with the importance and obligation of always telling the plain, unvarnished truth, by a discourse I heard from the noted Bishop Pierce. In his complete definition of a falsehood, he showed that to be guilty of this evil, it was not necessary to use words that were false in themselves, but intentionally making a wrong impression, no matter what was the language used, was really a falsehood, and just as damaging to character, or a cause, as a plain-spoken untruth. His remarks upon this question made so deep an impression upon me, as you say in regard to my last words to you, I have not forgotten them."

Mother—"It is difficult to estimate what good results sometimes follow from words that the speaker, perhaps, thinks have been but little heeded by those present, and for this reason I think we ought always to be careful of what we say in the presence of children, and all young people, as their plastic minds receive the most lasting impressions. I can remember when I was a child of hearing incidentally remarks made by older persons, the memory of which kept me often from committing wrong, or even coming in contact with it, after I grew towards womanhood. I am afraid we older people do not talk with as good effect in the presence of children now as they did when I was a child."

Doctor—"I don't think we do, madam. The truth is, in this pushing age, we have no time, it seems, for these good, old fashioned talks, that will instruct by making deep and lasting impressions. Every one is like I am this morning—in great haste to push on to something ahead; so we will get to business, by beginning where we left off at my last visit. Do you remember where that was?"

Mother—"Certainly I do, Doctor; we were speaking of lake or marsh water, which you said was entirely unfit for man or beast, and that you would give me your reasons for it when you called again."

Doctor—Yes, madam, such water is absolutely poisonous, because it is always impregnated with putrified vegetable and animal matter. As pure water is so essential to health, and can be obtained if the proper means are used, it is the imperative duty of every city and citizen to provide this great essential. It is now generally believed that impure water is one of the greatest causes of hog cholera."

Mother—"But, Doctor, how can we get pure drinking water in a city, especially where we have such a miserable, inefficient system of sewerage as in this place, and indeed in almost all of our southern cities? I asked this question at your last visit, but you did not have time to answer me."

Doctor—"Well, madam, the only available way I see at present is to have a properly constructed cistern on every house or lot, built so as to filtrate enough rain water for drinking purposes the year round. Where one has not the means to do this, and must use well or hydrant water, it can be purified to a great extent by boiling enough water every morning to last until the next, in a clean vessel into which nothing but water is ever put, then strain by dripping through a flannel bag. Put the water into bottles; cork tightly, and in the winter set in a cold place. In the summer put the bottles in a pail and keep enough ice around them to have the water pleasantly cold."

Mother (laughing)—"But, Doctor, how many, do you suppose, would be at that trouble to boil and filter the water? besides the poor could not afford the ice."

Doctor—"A good many could afford a little ice in the summer if they would do with less pork grease, and as to the trouble, it would not be near as great as to nurse perhaps several members of the family through an attack of typhoid fever, or malignant dysentery."

Mother—"No, I should think not. I am much obliged, Doctor, for this information about pure water, and, as I have said, we have been compelled to use all these kinds I mentioned, and now I shall always believe that several cases of typhoid fever in our fami'y were caused by the water we drank."

Doctor—" I have no doubt of it, madam; in my opinion, impure water and unsound food are the most pregnant causes of that type of fever."

Mother—" Well, I am glad Mary is now getting pure spring water, clear as crystal as it runs out of a rock and falls into a rocky basin. I have been to the spring at her uncle's, and I know *that* water is pure and wholesome. As to good, sound food, I have always thought before this, that Mary got that at home."

Doctor—" I know she gets the best the city affords, but you can get no article of food in the ctty as pure and wholesome as you can procure it in the country. Perhaps you will be surprised to hear, my dear madam, that milk produces a greater amount of disease than almost any article of food."

Mother—" You indeed surprise me. I thought milk was one of the best and most nourishing articles of food, and is so generally used, I never would have thought it productive of any disease."

Doctor—" Your surprise is very natural, madam, as it is only within the last five years that the attention of the medical scientist has been much directed towards the investigation of this great source of disease. The proof, though, is now abundant and conclusive, that milk adulterated with impure water, in many places, and particularly in large American and European cities, has been the cause of typhoid fever, diphtheria, diarrhœa and dysentery. It is also now generally believed that a large proportion of the deaths which occur among infants from bowel affections, is owing to milk thus tainted, and by putting it into nursing bottles that are seldom properly cleaned and kept in a pure atmosphere. Milk also becomes a medium of disease by absorbing poison from the air; therefore it should be closely covered, and never be kept in badly ventilated store-rooms with an unpleasant odor about them, and it should never be put in vessels made of lead or zinc. I have known several families poisoned by milk standing in such vessels, and in an impure atmosphere. Prof. Tyndall now asserts that diseases are propagated from solid particles discharged into the atmosphere by currents of air or gas."

Mother—"But how are we to know this, Doctor, and how did the Professor arrive at that conclusion?"

Doctor—"By the following experiment: He cut up a piece of steak, steeped it in water, heated it at a little above the temperature of the blood, then strained off the liquid. In a short time this fluid became turbid, and when examined through the microscope was found to be swarming with living organisms. By the application of greater heat, or by boiling, these organisms were killed, and when the solution was filtered he obtained a perfectly pure liquid, which, if kept free from particles of dust, would remain pure for an unlimited period; but he rejoined, that if a fly were to dip its leg in any fluid containing living organisms and then into a pure liquid, the whole of it would be swarming with animalcules in forty-eight hours."

Mother—"How wonderful! and is it not fortunate that people have a great repugnance to drinking anything into which one of these insects has fallen? Even one little fly may thus bring disease into a family."

Doctor—"Yes, and many of us, no doubt, drink milk, and broth, or soups into which the insect has not only fallen, but died there; but fortunately for our fastidiousness, and unfortunately for our health, we do not know it. Scarlatina has frequently prevailed in communities from drinking milk that had absorbed poison from the air. The truth of this mode of conveying disease is now so well established that in some sections of Europe the laws have instituted a regular scientific system for the inspection of even the cow-sheds, daries and everything pertaining to the milk that is used by the people. Unfortunately *our* people regard this as a small matter, especially in rapidly growing cities like Atlanta, where the parents cannot take time to eat themselves, much less to properly prepare the food and give it to their children in a manner and of a quality that will best conduce to their moral and physical development."

Mother—"You are rather hard on us of the far South, Doctor, but where were you raised, and where did you get your first start into the great physical proportions you have attained? Was it not in Virginia?"

Doctor—"My dear madam, you misapprehend me. I do not mean any reflection upon the people of your State, but simply to drop a good seed into the good soil I believe your mind to be, and indeed, I may say, the minds of women generally in Georgia, as I have found them, upon the whole, better educated than in almoet any State in the Union. You might have known from my avoirdupois and perfectly healthy appearance, that I was raised on a good bill of fare in Virginia, though there are a few similar specimens in your own State."

Mother—"I believe they do have most excellent food in Virginia, at least our Georgia soldiers who were quartered in that State spoke in the highest terms of its table hospitality, as well as its glorious patriotism. I believe, Doctor, that before we get through the subject of food, you will convince me that the proper selection and preparation of it accounts for Virginia being able to boast of having reared the largest number of the finest looking men and women in America."

Doctor—(laughing,) "I hope so, madam, and then you will, no doubt, understand how these fine men and women can be produced anywhere else, especially in Georgia, the progressive State of the South. But, I am glad to know that your daughter will get a plenty of good, sound food at her uncle's in the country. She will have milk pure and fresh; bread made from the best of wheat and corn. just from a water mill, and chickens that have not been imprisoned in coops reeking with foul odors; besides fresh game, mutton, choice fruit, etc. You know how those nice, motherly country ladies can feed any one at their tables."

Mother—"Yes, indeed, I have been at them often, and to hear you speak of them makes me want to go again. I did not intend for this interview to be so long, Doctor, but I find that the subject of food is equally important with that of drink, and in some respects is to me more interesting. I would be glad if you would enlighten me a little more in regard to our articles of daily food, and the disorders or diseases produced by them when not properly selected and prepared."

Doctor—"Well, madam, as I have already made some statements upon this subject, I will finish up that of meats, since that is an article of food upon which much intelligent and careful discrimination should be used. The most constant meat used in the city is that procured from the butcher. There are conflicting opinions in regard to the effects produced by eating unsound and diseased meats of this class. Epicures, generally, prefer game and fowls, also beef and mutton, cooked when the meat is on the verge of decomposition."

Mother—"I have known English people to pick their fowls, hang them up, and wait until they were in that state before they were cooked. They said it made the meat more tender, and more easily digested."

Doctor—Yes, I have known many such instances, and as it is common for the shepherds and farmers of Scotland to eat stale mutton from choice, and with impunity, such practices have given rise to the opinion with some, that diseased or decomposed meats are not poisonous as might be expected, or is supposed to be; but all agree that it is the thorough cooking of such meats that prevents them from causing dangerous or fatal diseases. I am convinced though, and there is an abundant proof to sustain me, that no matter how thoroughly such meats are cooked, there is a great risk to health, and sometimes life, in eating them."

Mother—"It is a risk to which I would never subject myself or family."

Doctor—"I have known many cases of vomiting, diarrhea, and a slow kind of fever produced by eating such food; also tape-worm, nettle rash, scurvey, and other diseases. These remarks will also apply to certain kinds of cured, or to bulk meat, and of which I have already spoken. All vegetable food that is unsound, or becoming mouldy, is also unwholesome; so is butter mixed with lard, vinegar made of sulphuric acid, pickles made green by adding sulphate of copper, and bread that is whitened by the use of alum, cake made light by the addition of ammonia, all of which food is bought and sold and eaten in our large towns and cities."

Mother—"What kind of bread, Doctor, do you think is the most wholesome, and very palatable besides?"

Doctor—"Well, madam, bread to be altogether wholesome, must first be made of meal or flour ground from perfectly sound corn or wheat, and where possible, fresh from a good water mill. Unbolt d flour is more wholesome than the white, but if the latter is used it should be made into bread after one or the other of the following directions. If light bread is desired, purely vegetable, or the old fashioned milk and salt yeast should be used, and so fresh that no carbonate of soda, or other alkali will be needed to correct the acid. But, if plain bread or "biscuit" is wanted, let it be prepared as our old mothers and grandmothers used to have it, by making it with no ingredients but good flour, salt, a little sweet lard, or butter, mix with water or sweet milk, and after sufficient kneading, have the sponge, or "dough" as some call it, well beaten on a block, then roll it thin as desired, cut it into biscuit, and bake brown in a slow oven. Such bread. eaten cold or moderately warm with a little fresh butter, is not unwholesome, and is palatable enough for an epicure."

Mother—"Yes, I have eaten it often at my mother's table, and know it is delicious."

Doctor—"When one can afford it, and the corn bread is desired, to be very good as well as wholesome, it can be made with perfectly sweet "clabber," as it is generally called, with no whey in it, then add sufficient melted butter to give the crust some crispness, then beat in as many eggs as you choose, one or more, according to quantity of batter, pour into a hot, buttered baking pan and cook immediately in a hot oven until brown, and well steamed. Corn meal "lightbread" that all these nice old-fashioned housekeepers know how to make, is also very good, and is wholesome. Where families cannot afford this more palatable process of making corn bread, they had better prepare it in the plain southern plantation manner of big, generous 'pones,' baked thoroughly and dry, in an old fashioned oven, or the equally good plain 'hoe-cake' and 'ash-cake.' I am satisfied that salaratus, tartarie acid, alum, ammonia and all these deleterious ingredients, are positively injurious, and finally destructive to health, and they are all employed to a great extent in the products of our bakeries, and also in private households."

Mother—"Judging from your remarks, the bread that most people eat in this country must have a great deal to do with the prevalence of ill health among them. This article of food is a very important one, is it not?"

Doctor—"There is none so much so, and it has been fitly called the staff of life. More bread is consumed than any other food; it is really the principal article of sustenance, and it is of vital importance to have it day by day of the best possible quality as regards the ingredients, the preparation of the bread and the manner of cooking it."

Mother—"I have observed that the almost universal manner in which white flour is prepared for bread, in both towns and the country, is what we call soda, or yeast powder biscuit."

Doctor—"Yes, and these soda biscuit are usually made yellow with the alkali, have a little rancid lard in them, are rolled, or made out two inches thick, and cooked about half done. Such bread, especially when eaten hot, and saturated with stale butter or some other grease, would demoralize the digestive organs of a mule, provided you could get the mule to eat it. No man, woman, or child can use this bread habitually without having the health destroyed sooner or later."

Mother—"But is not bread made of yeast powders wholesome?"

Doctor—"No, madam, even if you can get the article composed of pure materials, which you seldem, if ever, can do. Alkalies were intended by nature for mechanical, domestic, or medicinal purposes, and not as ingredients for regular food."

Mother—"All this indigestible food is an injury to the complexion as well as the health, is it not?"

Doctor—"Certainly, madam ; so long as a person has perfect digestion the complexion in a majority of cases will be clear and bright, with color in the cheeks and lips; the mind will be cheerful and active, and the body able to undergo fatigue that it would not under an opposite condition of the stomach."

Mother—"If our ladies, especially young girls, knew these things, they surely would be more careful about eating improper food. All women seem to set more value upon a beautiful complexion and fine teeth than any other personal attractions. That is why they use so many cosmetics."

Doctor—"All of which are ruinous to the complexion, and also, in nearly every case, injurious to the eyes and the general health. If ladies would only learn it, they would find that perfect health and cleanliness give a far more beautiful compl xion, and softer brightness to the eyes than any art can do. Good health and wholesome food, with proper care of the teeth, will preserve them also in their natural beauty. Hot food and drinks are injurious to the teeth as well as the stomach. But, madam, my time is now out, and must leave this subject for the present, and bid you good morning.

[TO BE CONTINUED.]

